# Partha Banerjea

**DOI:** 10.1192/pb.bp.114.046979

**Published:** 2014-04

**Authors:** Sabina Dosani

**Figure:**
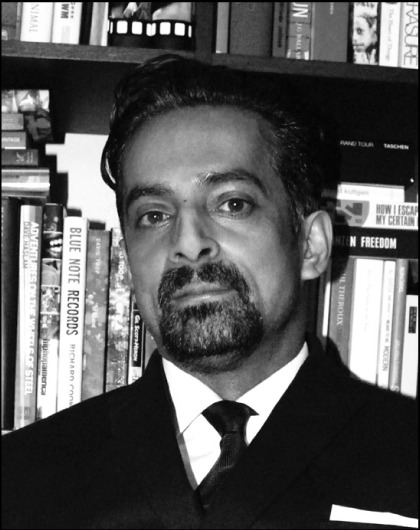


**Dr Partha Banerjea** is a consultant adolescent psychiatrist, running a high-risk, high-impact adolescent service at the Maudsley Hospital in South London. He is lead clinician for Southwark Child and Adolescent Mental Health Service, and visiting lecturer at the London School of Economics and Political Science. He has a keen interest in how to train future psychiatrists and interdisciplinary ideas such as the interface between psychiatry and the arts. He studied as an undergraduate at the University of Birmingham, then undertook postgraduate studies in New Zealand before returning to the Maudsley and St George’s to complete his training.

## What is your idea of a perfect mental health service?

Free at point of access, mindful of differential engagement patterns (race, ethnicity and class all play a part here), flexible, resourced financially with treatments that work, backed by research in the real world that utilises cross-disciplinary ideas as a way of embedding implementation, creative, and free from both central government whimsy and sanctimonious therapists making no difference in the lives of the people they respectfully serve and see.

## Which psychiatrist, living or dead, do you most admire?

Easily it would be Professor David Goldberg, who ran the adolescent service in Wandsworth while I was training. He is a psychiatrist who demonstrated immense generosity of spirit combined with a polymath’s ability to effortlessly synthesise academic data not only from psychiatry and numerous psychological therapies, but also from anthropology, sociology and politics, and he brought it alive often in the room with a patient, to their benefit. To this day it is unlikely ever to be surpassed.

## What do you consider to be your greatest achievements?

Teaching systemic theory through the films of Kurosawa, seeing the excitement in a trainee when a new idea sparks like a rewind in a dancehall, talking Miles Davies with colleagues and adolescents alike.

## What has been your most controversial idea?

That class may be a greater factor in alignments of perception than race and that if services wanted creativity they need to tolerate uncertainty and discomfort.

## What frustrates you most about working in psychiatry?

Government ministers introducing policy in stark contrast to available evidence, while simultaneously forgetting that they are here to serve the people. They forget that politics is showbiz for ugly people, and since most political careers end in failure, the price for their particular hubris should not be paid for by the everyman on the street.

The political manoeuvring that has cast us as managers of risk in a risk-averse culture that apportions blame while simultaneously stating that the structures within which we operate are blame free.

## Which phrases do you use most when speaking to trainees?

It’s a three-way tie between ‘The world is what it is’, a cornerstone of dialectical behaviour therapy, as a way of asking the trainee to hold change and stasis in mind at the same time (similar to Flaubert’s quote that contradiction is what keeps sanity in place), ‘What would Marx say?’ (both Karl and Groucho), shortly followed by ‘Have you done the Proust Questionnaire?’

## What single thing would improve the quality of your work?

Armbands so as to stop drowning in paperwork and an afternoon tea trolley serving Darjeeling and quality patisserie.

## What is the most important lesson that working in New Zealand taught you?

That life can be lived in many ways, outside of the many restrictions we self-impose, and that the quality of light changes how we perceive colour.

## What has been your biggest disappointment?

An equal tie between never having made it to the Paradise Garage and my continuing inability to conceal, while bowling, a decent doosra.

## What was the last book you read?

I tend to have three on the go, partly in the vain attempt to stimulate conversation between the ideas therein; currently, Turgenev’s *Fathers and Sons, Night Haunts* by Sukhdev Sandhu and James Astill’s *The Great Tamasha* are vying for my attention.

## How would you like to be remembered?

Similar to Leopold Bloom - he read his own obituary notice, so as to live longer; gives you second wind. New lease of life. Well, so they say.

